# Divergent metallothionein strategies underlie copper tolerance in *Saccharomyces* species relevant to winemaking

**DOI:** 10.1007/s00253-026-13869-z

**Published:** 2026-05-21

**Authors:** Raquel Sorribes-Dauden, David Peris, María Teresa Martínez-Pastor, Sergi Puig

**Affiliations:** 1 Departamento de Biotecnología, Instituto de Agroquímica y Tecnología de Alimentos (IATA), Consejo Superior de Investigaciones Científicas (CSIC), 46980 Paterna, Valencia, Spain; 2 FunGIALab, Department of Biosciences, University of Oslo, 0371 Oslo, Norway; 3 Departamento de Bioquímica y Biología Molecular, Universitat de València, 46100 Burjassot, Valencia Spain

**Keywords:** Copper, Wine, *Saccharomyces*, Metallothioneins, Cup1, Crs5

## Abstract

**Abstract:**

Copper is an essential redox-active metal that serves as a cofactor in fundamental biological processes, including mitochondrial respiration and iron homeostasis. However, excess copper promotes the formation of reactive oxygen species (ROS), leading to cellular damage and death. Owing to its antimicrobial properties, copper is widely used in winemaking. *Saccharomyces cerevisiae*, the primary fermentative yeast, responds to copper excess by transcriptionally inducing the expression of metallothioneins *CUP1* and *CRS5*, along with antioxidant defenses. In this study, we examined copper tolerance across the *Saccharomyces* genus. Consistent with previous work, *CUP1* copy number variation (CNV) and expression were the main determinants of copper tolerance in wine *S. cerevisiae* strains. In contrast, wild *Saccharomyces* species, harboring only a single *CUP1* copy, showed no correlation between copper tolerance and *CUP1* expression levels. Notably, species within the *Saccharomyces* clade closely related to *S. cerevisiae* predominantly expressed *CUP1*, whereas more distantly related species preferentially expressed *CRS5*. These findings highlight a substantial role for Crs5 metallothionein in copper detoxification when *CUP1* amplification is absent and suggest that additional Cup1-independent mechanisms contribute to copper tolerance in non-domesticated *Saccharomyces* species.

**Key points:**

- *Copper-sensitive wine yeasts exhibit increased reliance on Crs5 metallothionein.*
- *Saccharomyces species closely related to S. cerevisiae preferentially express CUP1.*
- *Saccharomyces species distantly related to S. cerevisiae predominantly express CRS5.*

**Supplementary Information:**

The online version contains supplementary material available at 10.1007/s00253-026-13869-z.

## Introduction

Copper is a redox metal that participates as a cofactor in biological processes as diverse as cellular respiration, iron metabolism, melanin biosynthesis, connective tissue formation, neurotransmitter synthesis, and photosynthesis (Festa and Thiele 2011). However, when present in excess, copper can engage in Fenton-like reactions and Haber–Weiss cycles to generate reactive oxygen species (ROS) that damage cells at the level of proteins, nucleic acids, and lipids (Halliwell and Gutteridge 1984). Recent studies have uncovered a regulated cell death program denoted cuproptosis that is dependent on copper ions (Tsvetkov et al. 2022). For these reasons, copper is used in multiple antimicrobial applications including agriculture control of plagues, surface coating in hospital devices or high-touch surfaces, and water disinfection.

In viticulture and enology, copper plays a key role for disease control and wine quality mainly through its antimicrobial and chemical properties (Lamichhane et al. 2018). Copper-based compounds, like the Bordeaux mixture (copper sulfate and calcium oxide), are widely applied to limit fungal diseases such as downy and powdery mildew, caused by *Uncinula necator* and *Plasmopara viticola,* respectively, in vineyards. Copper sulfate and copper citrate are also used in winemaking to remove hydrogen sulfide and other sulfur compounds that cause off-odors. Copper in grape must can inhibit microbial growth during fermentation. Thus, microorganisms exposed to high copper concentrations have to develop adaptation mechanisms such as efflux, sequestration or storage systems, cell envelope modification, activation of antioxidant defenses, or biofilm formation, to survive copper toxicity (Arguello et al. 2013; Shi et al. 2021).

The budding yeast *Saccharomyces cerevisiae* is the principal organism responsible for fermenting grape juice into wine. In response to high copper levels, the *S. cerevisiae* copper-sensing transcription factor Ace1, also known as Cup2, activates the expression of (i) the metallothioneins *CUP1* and *CRS5*, (ii) the copper/zinc superoxide dismutase *SOD1*, and (iii) the high-affinity cell-surface iron uptake system *FET3*/*FTR1* (Culotta et al. 1994; Fay et al. 2004; Gaspar-Cordeiro et al. 2018; Gralla et al. 1991; Gross et al. 2000; Thiele 1988; Welch et al. 1989). *CUP1* and *CRS5* are non-homologous metallothioneins that bind and detoxify excess metal ions (Butt et al. 1984; Culotta et al. 1994; Karin et al. 1984; Pagani et al. 2007). According to their Cu(I)- and Zn(II)-binding preferences, Cup1 can be classified as a genuine copper-thionein whereas Crs5 is considered to have an intermediate character (Pagani et al 2007; Palacios et al 2011). Sod1 neutralizes superoxide radicals and contributes to copper buffering (Culotta et al. 1995). It has been proposed that the enhanced iron acquisition mediated by the Fet3/Ftr1 complex attenuates the activation of the iron-regulated transcription factor Aft1, thereby decreasing copper uptake through low-affinity metal transporters (Gaspar-Cordeiro et al. 2018). Besides, copper toxicity does not only involve metal detoxification systems but also triggers a strong oxidative stress response**.** Among the oxidative defense mechanisms, cytosolic thioredoxin Trx2 plays a central role in maintaining thiol and redox homeostasis, and contributes to yeast stress tolerance during winemaking (Kuge and Jones 1994; Picazo et al. 2019).

Wine/European strains isolated from vineyards and wine fermentations, as well as sake and clinical isolates, display higher copper resistance than wild *S. cerevisiae* strains, indicating that copper tolerance is a trait shaped by domestication and adaptation (Fay et al. 2026; Gallone et al. 2016; Strope et al. 2015; Warringer et al. 2011). The primary mechanism underlying adaptation to elevated copper concentrations in *S. cerevisiae* involves tandem amplification of the *CUP1* locus (Fogel and Welch 1982; Hamer et al. 1985), whereas no amplification of *CRS5* has been reported (Adamo et al. 2012). Studies using a laboratory *S. cerevisiae* strain have shown that *CUP1* protects cells from copper toxicity with enhanced effectiveness than *CRS5*, owing both to its stronger transcriptional induction by copper and to a higher copper-binding affinity of the Cup1 protein (Jensen et al. 1996).

Despite also being isolated in vineyards, strains from the closest *S. cerevisiae* relative *Saccharomyces paradoxus* exhibit low copper tolerance and do not exhibit amplification of the *CUP1* locus (Dashko et al. 2016; Liti et al. 2009; Longan and Fay 2024; Warringer et al. 2011). The aim of this study is to extend the investigation of copper tolerance across the *Saccharomyces* genus. We hypothesize that, although *CUP1* copy number and expression are the primary determinants of copper tolerance in wine *S. cerevisiae* strains, additional factors, such as the metallothionein *CRS5*, may also contribute to copper resistance in non-domesticated *Saccharomyces* strains. To evaluate this hypothesis, we combined comparative genomic and gene expression analyses using available assembled *Saccharomyces* genomes with phenotypic profiling, ROS quantification, and cell viability measurements.

## Materials and methods

### Yeast strains and growth conditions

All the strains from the different *Saccharomyces* species utilized in this work are shown in Supplemental Table S1. Yeasts were stored and maintained as previously described (Sorribes-Dauden et al. 2022). To achieve similar growth rates for all *Saccharomyces* species under standard conditions in synthetic complete medium (SC), cultures were grown at 20 °C (Peris et al. 2023). Copper sulfate (CuSO_4_·5H_2_O; Panreac, Barcelona, Spain) from a 200 mM stock solution in Milli-Q water (Millipore, MA, USA) was added to SC liquid medium at the indicated concentrations.

### Determination of non-inhibitory and minimal inhibitory concentrations

To determine the non-inhibitory concentration (NIC) and minimal inhibitory concentration (MIC), yeasts were inoculated in 96-well plates, growth was recorded as optical density at 600 nm (OD_600_) in a microplate reader equipped with a stacker-based plate handling system, Spectrostar Omega (BMG Labtech, Ortenberg, Germany), and the results analyzed as previously described (Sorribes-Dauden et al. 2022).

### Genomic DNA extraction

Overnight precultures were grown in YPD (1% yeast extract, 2% peptone, 2% glucose), and genomic DNA (gDNA) was extracted as previously described (Querol et al. 1992) with a few modifications. Briefly, harvested cells were resuspended in 500 µL buffer I (0.9 M sorbitol, 0.1 M ethylenediaminetetraacetic acid (EDTA) and 10 mg/mL zymolyase 20 T; Merck-Sigma, Darmstadt, Germany) and incubated at 37 °C for 30–60 min. The resulting spheroplasts were resuspended in 500 µL of buffer II (50 mM Tris–HCl pH 7.4, 20 mM EDTA, and 10% sodium dodecyl sulfate (SDS)), and the mixture was incubated at 65 °C for 5 min. Then, 200 µL of 5 M potassium acetate were added, and tubes were put on ice for 30 min Lysates were centrifugated at 4 °C for 15 min at 14,000 rpm, and gDNA was precipitated with 700 µL isopropanol (1:1, v:v) for 10 min at room temperature and centrifuged at 4 °C for 15 min at 14,000 rpm. gDNA was washed twice with 70% ethanol, vacuum dried, and dissolved in Milli-Q water (Millipore, MA, USA). Finally, RNA was removed by treatment with 4 µL of RNase A (1 mg/mL; Merck-Sigma, Darmstadt, Germany) for 30 min at 37 °C.

### Determination of *CUP1* copy number variation (CNV) from gDNA

Quantitative PCR (qPCR) analyses were performed using a LightCycler 480 II system (Roche, Basel, Switzerland) and the SYBR Premix Ex Taq kit (TaKaRa, Kusatsu, Japan) for fluorescent detection, following the protocol described by Sanvisens et al. (2014) with adaptations for gDNA analyses. The thermal cycling conditions were as follows: an initial denaturation at 95 °C for 10 s, followed by 40 cycles of denaturation at 95 °C for 10 s, and annealing at 55 °C for 15 s. A standard curve was generated using serial dilutions (1/5, 1/10, 1/50, 1/100, 1/500, and 1/1000) of a gDNA pool derived from a mixture of all strains. Species-specific primers are listed in Supplemental Table S2. *ACT1* gene copy number was used for normalization, and the DTY3 strain (Supplemental Table S1) was used as the reference for a single-copy gene signal.

### Determination of *CUP1* CNV from sequence analysis

Genome assemblies were obtained from GenBank (accessions in Supplemental Table S1), our *Sac2.0* GitHub repository (https://perisd.github.io/Sac2.0/), or from the repositories referenced in the original publications when GenBank accessions were unavailable (Supplemental Table S1). *CUP1* gene quantification was performed after annotation using BLASTx, with the *CIC1*–*RSC30* region—which contains *CUP1* sequences—used as the query.

### Cellular viability and oxidative stress measures

Overnight precultures were inoculated at an OD_600_ of 0.2 in fresh SC liquid media in glass flasks and grown until exponential phase was reached (~ 1.0 OD_600_/mL). After 14 h of incubation with 5 mM copper sulfate, an aliquot was taken for propidium iodide (PI, Merck-Sigma, Darmstadt, Germany) and dihydrorhodamine 123 (DHR 123, Merck-Sigma, Darmstadt, Germany) labelling as previously described with minor modifications (Bisquert et al. 2018). PI and DHR 123 were added separately to the cultures at a final concentration of 5 μg/mL, using 1 mg/mL and 2.5 mg/mL stock solutions prepared in Milli-Q water (Millipore, MA, USA) and ethanol, respectively. Then, cells were incubated in the dark for 15 min (PI) and 90 min (DHR 123) and analyzed using the “Annexin V and Cell Death” channel of the flow cytometer Muse Cell Analyzer (Millipore, MA, USA). The settings were adjusted using positive (15 min at 60 °C) and negative (non-stressed cells) controls. Data are presented as the percentage of cells exhibiting PI or DHR 123-positive staining and represent the mean and standard deviation of three replicates.

For each combination of fluorophore type (PI or DHR 123) and time (14 and 18 h), a one‑way analysis of variance (ANOVA) was performed to assess whether fluorescence levels differed significantly among strains. Post‑hoc pairwise comparisons were performed using Tukey’s Honestly Significant Difference test (Tukey HSD test) to identify which strains differed significantly from each other at the two different time points. All analyses were performed in RStudio 2025.09.0 + 387 (R Core Team 2025).

### RNA extraction and analysis

Overnight precultures were reinoculated in SC liquid medium and grown in glass flasks overnight until cells reached exponential phase. Once cells achieve an OD_600_ of approximately 1.0; they were exposed to 5 mM copper sulfate for 1 h. Cells were harvested, and total RNA extracted and analyzed by RT-qPCR, as previously described (Sanvisens et al. 2014). Strain-specific primers used for RT-qPCR are listed in Supplemental Table S2. The *ACT1* mRNA values were used to normalize.

### Gene expression heatmaps

The heatmap analysis was performed using the *pheatmap* R package (v. 1.0.13; (Kolde 2025)) within RStudio 2025.09.0 + 387 (R Core Team 2025). Hierarchical clustering was based on Euclidean distance, and *Z*-score normalization was applied for each strain or gene, as indicated in figure legends.

### Principal component analyses (PCA)

PCA was obtained in R Studio 2025.09.0 + 387 (R Core Team 2025), using the *prcomp* function of *factoextra* package v. 1.0.7 (Kassambara and Mundt 2020). PCA components histogram and biplot were generated using *fviz_eig* and *fviz_pca_biplot* tools from *ggfortify* package v. 0.4.19 (Tang et al. 2016).

### Scatterplot analysis

Scatterplot analysis was performed using the *ggplot2* R package (v. 4.0.0; (Wickham 2016)) within RStudio 2025.09.0 + 387 (R Core Team 2025). Correlation coefficients and regression equations were calculated using Spearman’s correlation method.

## Results

### Characterization of copper tolerance in species from the genus *Saccharomyces*

**Fig. 1:**
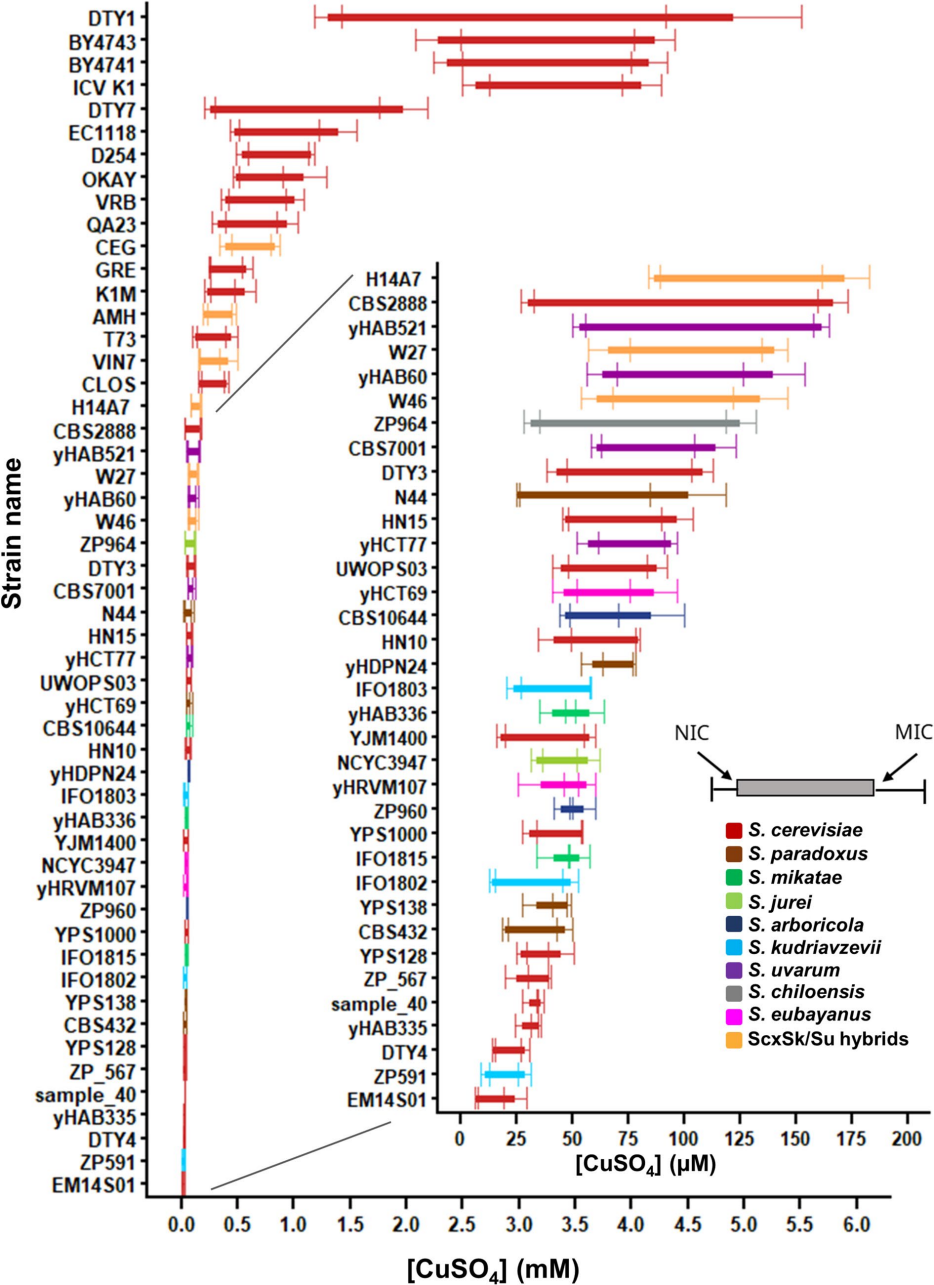
Copper tolerance of *Saccharomyces* yeasts. Bar plots representing the NIC (left-end of the bar) and MIC (right-end of the bar) average values (*n* = 3 independent biological replicates) for CuSO₄. Error bars represent the standard deviation, the left for the NIC and the right for the MIC. Species are colored according to the legend

Copper homeostasis has primarily been investigated in specific strains of *S.* *cerevisiae*. To gain broader insight into how yeasts adapt to elevated copper levels, we assessed copper tolerance across multiple species within the *Saccharomyces* genus. We analyzed 52 strains representing *S. cerevisiae*, *S. paradoxus*, *Saccharomyces* *mikatae*, *Saccharomyces* *kudriavzevii*, *Saccharomyces* *arboricola*, *Saccharomyces* *eubayanus*, *Saccharomyces* *uvarum*, the recently described *Saccharomyces* *chiloensis* (Pena et al. 2024) from Australasia population (Peris et al. 2023), and hybrids of *S. cerevisiae* with *S. uvarum* or *S. kudriavzevii* (see Supplemental Table S1 for strain details). Strains were grown in synthetic complete (SC) liquid medium supplemented with increasing concentrations of copper sulfate, and both the NIC and MIC values were determined (Fig. 1). Our results show that wine *S. cerevisiae* strains and hybrids exhibit markedly higher NIC and MIC values compared to wild *Saccharomyces* species, including non-domesticated *S. cerevisiae* strains, indicating greater copper tolerance. Except for *S. cerevisiae* × *S. kudriavzevii* hybrids W27 and W46, most wine-related strains display MIC values between 0.5 and 4 mM CuSO₄, whereas non-domesticated strains exhibit MIC values ranging from 25 to 200 μM CuSO₄ (Fig. 1). Furthermore, the range of concentrations between NIC and MIC values is larger in wine isolates. These findings are consistent with wine yeast strains adapting to the elevated copper concentrations commonly encountered in vineyards and grape musts.

### *CUP1* copy number variation predicts copper tolerance in wine yeasts, but not in wild *Saccharomyces* yeasts

**Fig. 2:**
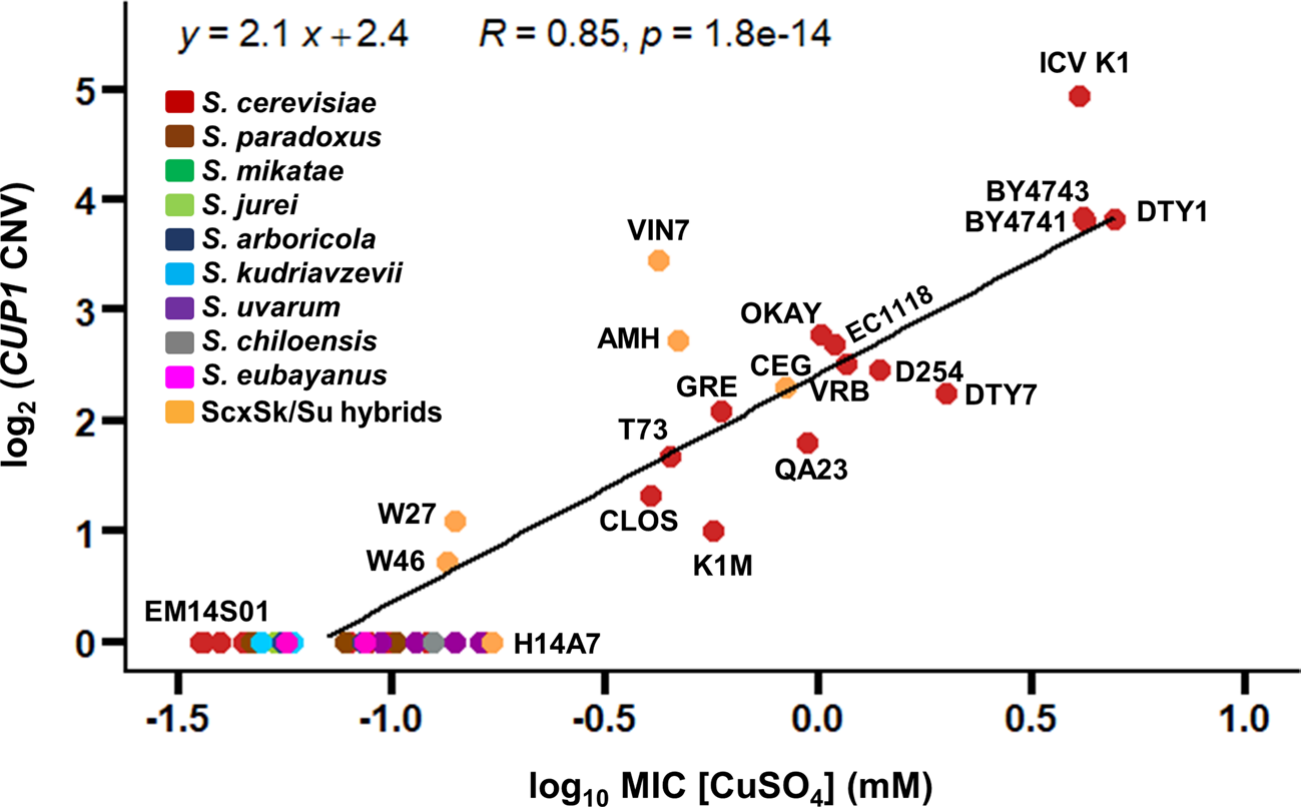
*CUP1 *copy number does not fully explain yeast copper tolerance. Scatterplot analysis of log_10_ CuSO_4_ MIC (*x* axis) and log_2_ *CUP1* CNV (*y* axis). Spearman’s correlation equation, correlation coefficient (*R*), and *p* value (*p*) are shown. Dots were colored according to the species designation displayed in the legend

A primary determinant of copper tolerance in *S. cerevisiae*, contributing to ~ 50% of copper resistance, is the copy number variation (CNV) of the *CUP1* gene (Adamo et al. 2012; Fogel and Welch 1982; Hamer et al. 1985; Peter et al. 2018; Strope et al. 2015; Warringer et al. 2011; Yue et al. 2017). To assess whether *CUP1* CNV also influences copper tolerance across the *Saccharomyces* genus, we used qPCR to quantify *CUP1* gene copies in *S. cerevisiae* strains and hybrids, and *CUP1* gene annotation in assembled genomes for non-*cerevisiae* strains (Supplemental Table S1). Across all non‑*cerevisiae Saccharomyces* species, *CUP1* was consistently present as a single copy, retaining the same local synteny observed in the laboratory BY4743 strain. Notably, *S. eubayanus, S. uvarum*, and *S. chiloensis* preserved this syntenic arrangement, but they carried a previously described chromosomal translocation placing *CUP1* on chromosome XV (Baker et al 2015; Nespolo et al 2020; Scannell et al 2011; Supplemental Table S1). The DTY3 strain, which carries a single *CUP1* copy, served as a reference, and values were normalized to the copy number of the *ACT1* gene in each strain. We observed that wine yeast strains exhibited *CUP1* copy numbers ranging from 2 to 30, whereas wild strains consistently carried a single copy of *CUP1*, which agrees with *CUP1* quantification at genome level (Supplemental Table S1). Comparison of *CUP1* CNV with copper tolerance (MIC values) revealed a robust linear correlation in wine strains (*R* = 0.85; *p* < 1.8 × 10^−14^), while non-domesticated strains showed variable copper tolerance despite possessing only one *CUP1* metallothionein gene (Fig. 2). These results confirm previous findings indicating that *CUP1* copy number is a major determinant of copper tolerance in wine *Saccharomyces* strains (Adamo et al. 2012; Fogel and Welch 1982; Hamer et al. 1985; Peter et al. 2018; Strope et al. 2015; Warringer et al. 2011; Yue et al. 2017), but they suggest that additional factors likely contribute to copper resistance in *Saccharomyces* species with a single copy of *CUP1* gene.

### Characterization of oxidative stress and cell death in *Saccharomyces* yeasts with different copper sensitivities

**Fig. 3:**
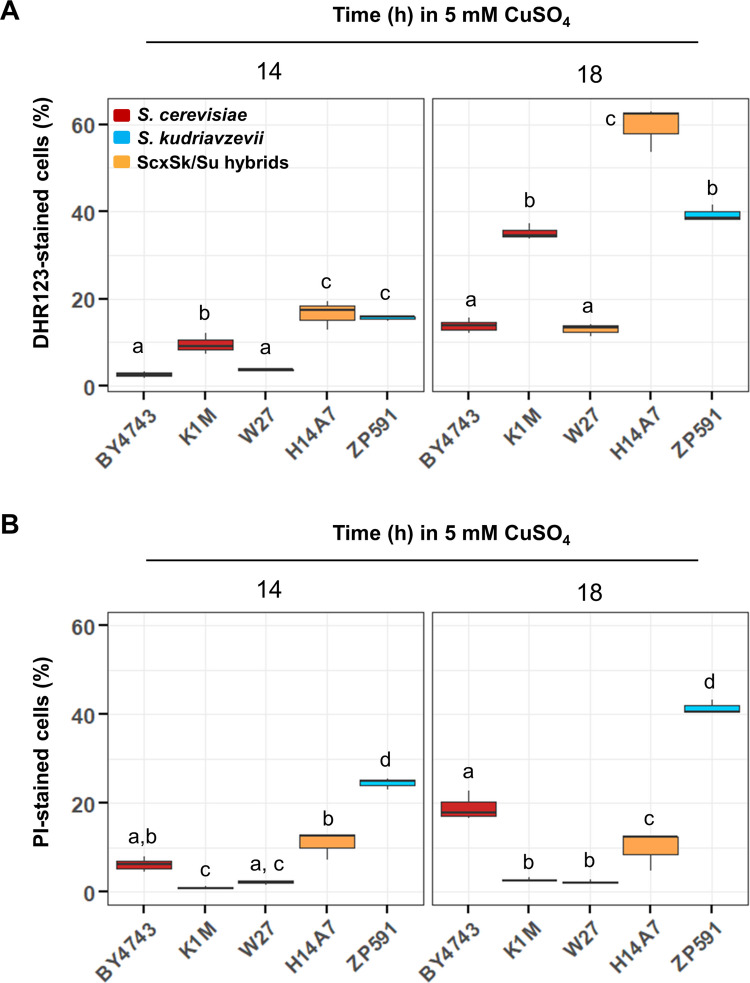
Oxidative stress and cell death in yeasts with different copper sensitivity. ROS production (**A**) and cell death (**B**) were determined using flow cytometry with DHR 123 staining and PI, respectively, after 14 and 18 h of 5 mM CuSO_4_ exposure. Boxplots indicate the interquartile range (IQR), with horizontal lines indicating medians and whiskers extending to 1.5 × IQR. Post‑hoc pairwise comparisons between strains were performed using Tukey HSD test. Identical letters denote groups that are not significantly different (*p* > 0.05) (Supplemental Table S3)

Excess copper promotes the generation of ROS, which oxidizes cellular components and ultimately leads to cell death (Halliwell and Gutteridge 1984). To determine whether copper sensitivity correlates with oxidative stress, we quantified the oxidation of dihydrorhodamine 123 (DHR 123) to its fluorescent product, rhodamine 123, by flow cytometry. We analyzed three yeasts carrying more than one copy of *CUP1*, representing the most tolerant (BY4743), the least tolerant (W27), and an intermediate phenotype (K1M), as well as two strains harboring a single copy of *CUP1*, H14A7, and ZP591, which represent the most and least tolerant among the single-copy strains, respectively. Cultures were exposed to 5 mM copper sulfate for 14 and 18 h. Single-copy *CUP1* yeasts exhibited significantly higher ROS levels than strains carrying multiple *CUP1* copies at both 14 and 18 h (Fig. 3A). After 18 h, strains with multiple *CUP1* copies reached ROS levels comparable or higher than those shown by single-copy *CUP1* yeasts at 14 h (Fig. 3A). Surprisingly, the copper tolerant strain K1M reached at 18-h ROS amounts that were not statistically different from those observed in the copper-sensitive strain ZP591 (Fig. 3A).

To assess cell viability, we performed propidium iodide (PI) staining, which selectively penetrates cells with compromised membranes, followed by flow cytometric analysis. As expected, the more copper-sensitive single-copy *CUP1* yeasts, H14A7 and ZP591, displayed higher levels of cell death values than the more copper-tolerant yeasts K1M and W27, with multiple *CUP1* genes (Fig. 3B). ZP591, the most copper-sensitive yeast, showed the highest proportion of PI-positive cells (Fig. 3B). Unexpectedly, the laboratory *S. cerevisiae* strain BY4743 exhibited substantial cell death, despite being the most copper-tolerant strain, possessing the highest *CUP1* copy number, and showing the lowest ROS levels (Fig. 3). Conversely, the relatively copper-tolerant K1M yeast showed minimal cell death despite exhibiting relatively elevated ROS values (Fig. 3). Taken together, these results suggest that yeasts with increased *CUP1* copy number generally display lower oxidative stress and reduced cell death under copper exposure. However, the discrepancies among some yeasts, particularly BY4743 and K1M, suggest that other factors beyond *CUP1* dosage and oxidative stress influence cell survival in high-copper conditions.

### Copper-sensitive wine yeasts exhibit increased reliance on Crs5 metallothionein

**Fig. 4:**
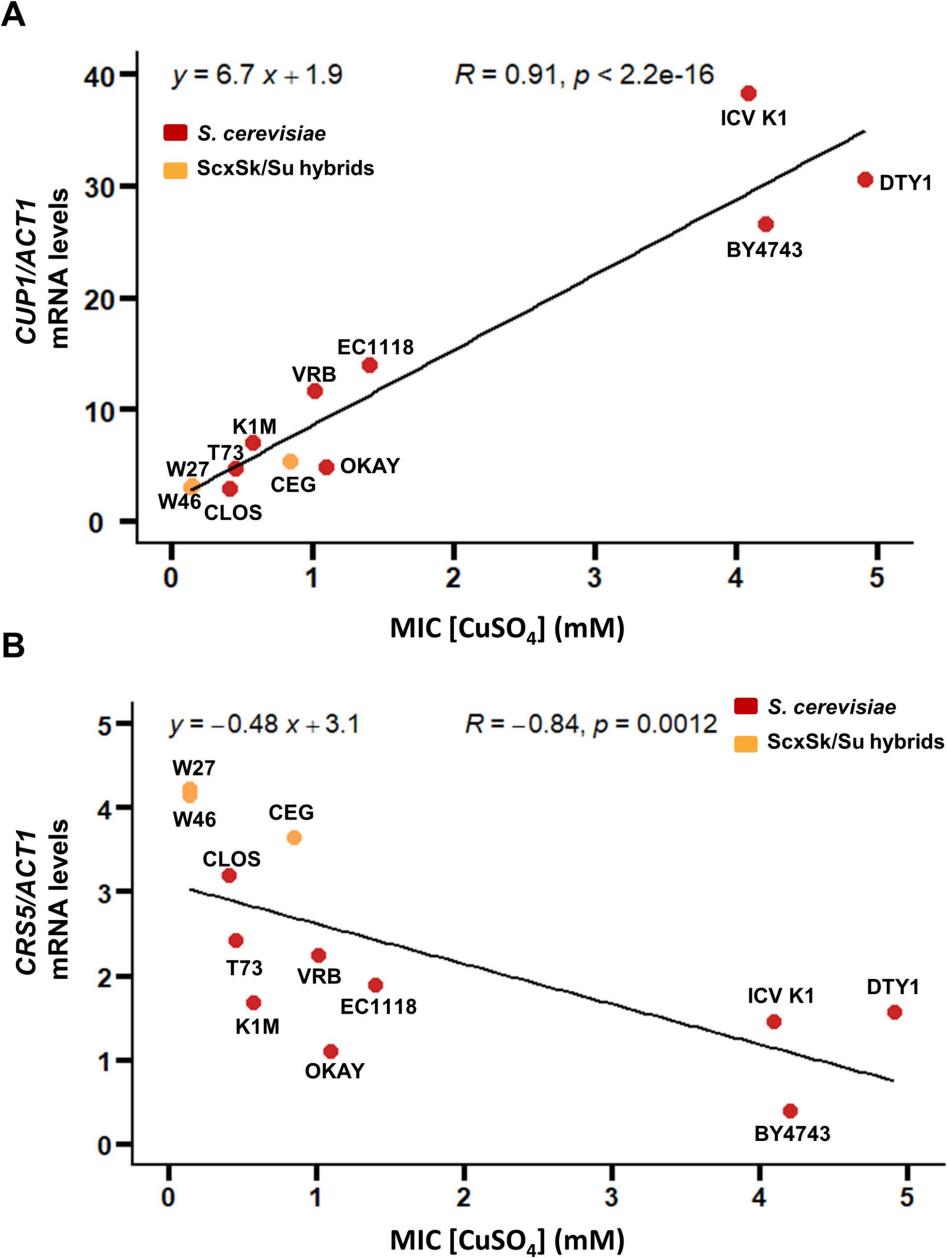
Correlation between metallothionein expression and copper tolerance in wine yeasts. Scatterplot analysis of log_10_ MIC CuSO_4_ values (*x* axis) and *CUP1* (**A**) or *CRS5* (**B**) mRNA levels after 1 h of exposure to 5 mM CuSO_4_ (*y* axis) in wine yeasts with more than one *CUP1* copy. Data represent the average of three independent biological replicates and is normalized to *ACT1* mRNA levels in the same conditions (*y* axis). Spot colors follow the same code as previously. Spearman’s correlation equation and correlation coefficient (*R*) are shown, together with *p* value (*p*)

To quantify *CUP1* expression in wine yeasts, we explored different growth conditions, including multiple copper concentrations and times of exposure, to conclude that 5 mM CuSO₄ for 1 h led to the best induction, as previously reported (Peña et al. 1998). Transcript abundance was assessed by RT-qPCR and normalized to *ACT1* mRNA levels (see Materials and methods). As expected, in wine yeasts, *CUP1* transcript levels displayed a strong linear correlation with *CUP1* CNV (Supplemental Fig. S1; *R* = 0.83; *p* < 0.001). Furthermore, a significant positive association was observed between *CUP1* mRNA levels and MIC values across wine yeasts (Fig. 4A and Supplemental Fig. S2A; *R* = 0.91; *p* value < 2.2 × 10^−16^), indicating that copper-tolerant isolates exhibit markedly higher *CUP1* expression.

To further characterize the transcriptional response of the different wine *Saccharomyces* yeasts under high-copper conditions, we examined the expression of the *CRS5* copper metallothionein gene together with the oxidative‑stress response genes *SOD1* and *TRX2*. Although Crs5 contributes to copper detoxification by sequestering intracellular copper (Culotta et al. 1994), we observed that *CRS5* transcript abundance exhibited an inverse relationship with copper tolerance among wine yeasts (Fig. 4B and Supplemental Fig. S2B). Specifically, highly tolerant strains, such as ICV K1, DTY1, and BY4743, displayed elevated *CUP1* mRNA levels but markedly reduced *CRS5* expression, whereas the opposite pattern was observed in the copper-sensitive strains W27 and W46 (Fig. 4). No significant correlation was apparently detected between copper tolerance and the expression of either *SOD1* or *TRX2* genes (Supplemental Figs. S2 and S3).

**Fig. 5:**
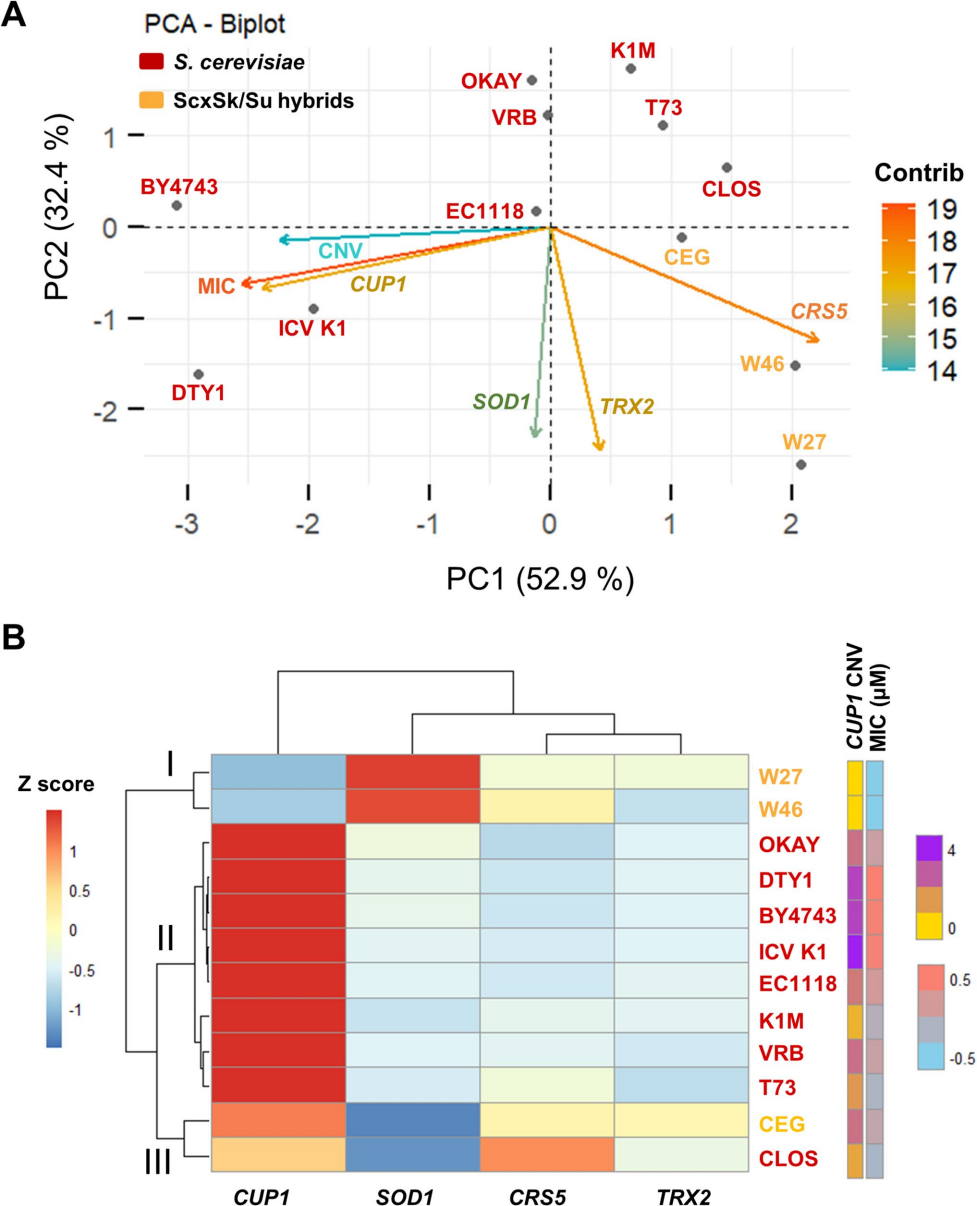
Expression of copper and oxidative stress genes in wine strains.** A** PCA biplot displays principal components 1 (PC1, 52.9%) and 2 (PC2, 32.4%), with the contribution (contrib) of each variable indicated by arrow length and color. Light blue to green colors represents low contributions, whereas orange to red colors indicate high contributions of the variable to the variance. Dots represent strains, which distribution reflects the diversity of responses to copper. Dot names are colored according to the legend. ScxSk/Su: *S. cerevisiae* x *S. kudriavzevii*/*S. cerevisiae* x *S. uvarum*. **B** Heatmap analysis of C*UP1*,* CRS5*,* SOD1*, and *TRX2* mRNA levels relative to *ACT1* transcript levels. Hierarchical clustering was performed based on Euclidean distance, and groups I, II, and III indicate the resulting clusters. *Z*-score normalization was performed for each strain. High values are shown in red, whereas low values are shown in blue. Tolerance is depicted as log₁₀(MIC), with high values shown in salmon pink and low values in light blue. log₂ (CNV) values are represented in purple (high) and yellow (low)

A PCA integrating absolute expression levels of the four genes, MIC values, and *CUP1* CNV, revealed *CUP1* expression as the dominant contributor to variance, effectively separating the highly copper-tolerant strains ICV K1, DTY1, and BY4743 from the remaining isolates (Fig. 5A and Supplemental Fig. S4A). *CRS5* expression also significantly contributed to the discrimination of copper-sensitive strains, notably W27 and W46, from the other groups (Fig. 5A). To further explore these patterns, relative expression profiles of the four genes were jointly analyzed, revealing three distinct clusters. The largest group II comprised copper-tolerant *S. cerevisiae* yeasts characterized by markedly elevated *CUP1* expression relative to the other genes, whose transcript levels remained low (Fig. 5B and Supplemental Fig. S4B). In contrast, groups I and III, which included copper-sensitive yeasts such as *S. cerevisiae* CLOS and the hybrids *S. cerevisiae* × *S. kudriavzevii* W27 and W46, exhibited reduced *CUP1* expression but comparatively higher *CRS5* transcript abundance (Fig. 5B). Notably, *SOD1* expression also varied among groups: the most sensitive strains (group I) displayed the highest *SOD1* transcript levels, which might indicate a higher induction of the response against oxidative stress, whereas group III exhibited the lowest (Fig. 5B). Collectively, these findings indicate that copper-tolerant wine yeasts primarily rely on *CUP1* expression for detoxification, whereas copper-sensitive wine yeasts seem to depend more heavily on Crs5 metallothionein.

### Other mechanisms beyond *CUP1* expression contribute to copper tolerance in non-domesticated *Saccharomyces* species

**Fig. 6:**
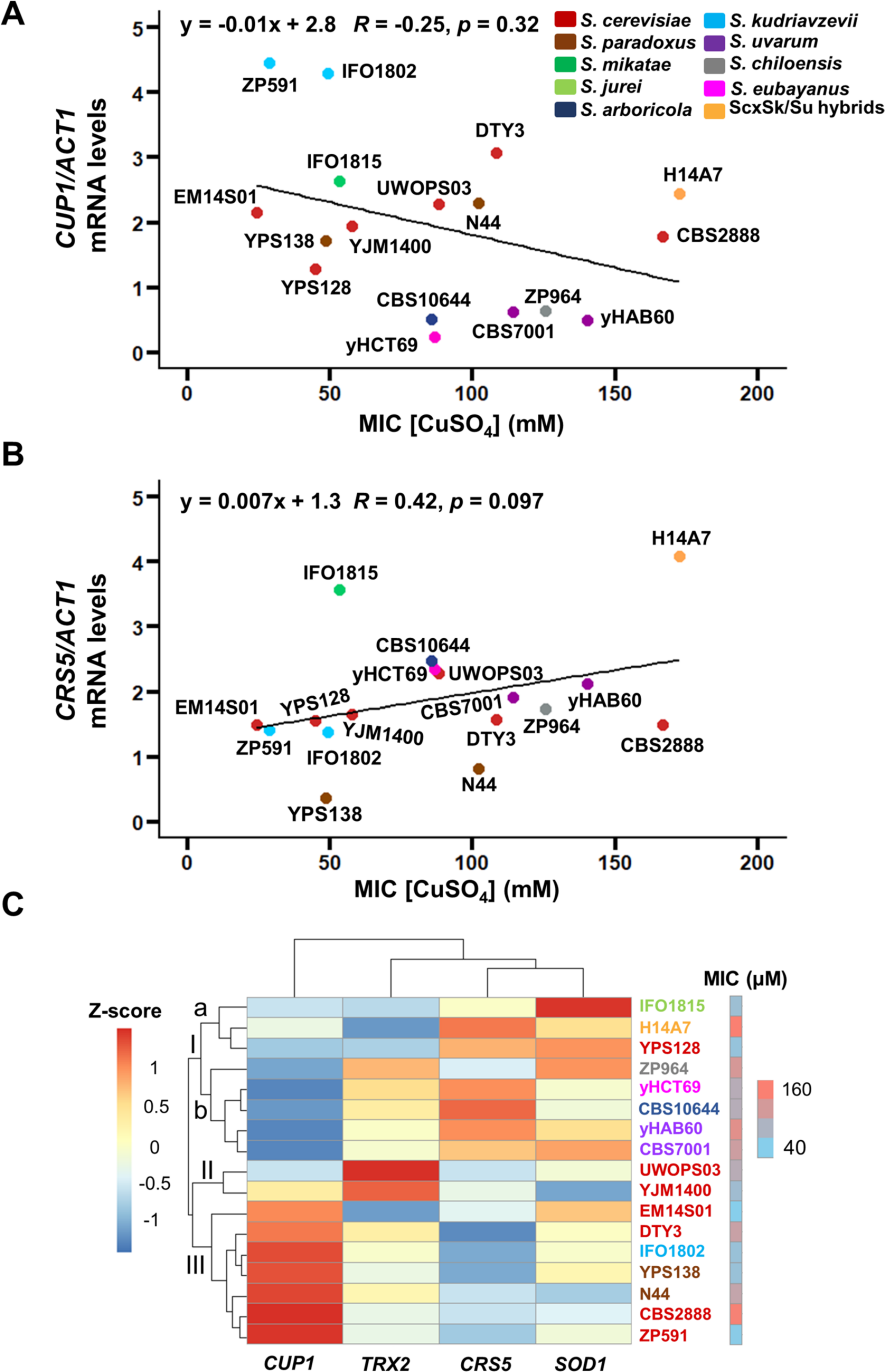
Expression of *CUP1* and *CRS5* in strains carrying a single *CUP1 *copy. Scatterplot analysis of CuSO_4_ MIC (*x* axis) and *CUP1* (**A**) or *CRS5* (**B**) mRNA levels under 1 h of exposure to 5 mM CuSO_4_ (*y* axis) in strains with one *CUP1* copy. Data represent the average of three independent biological replicates and is normalized to *ACT1* mRNA levels in the same conditions (*y* axis). Spot colors follow the same code as previously. Spearman’s correlation equation and correlation coefficient (*R*) are shown, together with *p* value (*p*). **C** Heatmap analysis of C*UP1*, *CRS5*,* SOD1*, and *TRX2* mRNA levels relative to *ACT1* mRNA levels after 1 h of exposure to CuSO_4_. Hierarchical clustering was performed based on Euclidean distance; resulting groups Ia, Ib, II, and III are highlighted. *Z*-score normalization was performed for each strain, and high values are shown in red, whereas low values are shown in blue. Tolerance is depicted as log₁₀(MIC), with high values shown in salmon pink and low values in light blue

Strains of *S. cerevisiae* unrelated to winemaking, as well as other *Saccharomyces* species, harbor only a single copy of the *CUP1* gene despite displaying variable sensitivity to environmental copper (Supplemental Table S1 and Fig. 1). To explore the molecular basis of this variation, we quantified the expression of *CUP1*, *CRS5*, *SOD1*, and *TRX2* genes, as performed for wine yeasts. No clear correlation was observed between the expression levels of these genes (including *CUP1*) and copper tolerance (Fig. 6 and Supplemental Fig. S5). Thus, unlike wine yeast strains with multiple *CUP1* copies, copper tolerance in wild strains cannot be explained solely by *CUP1* expression. For example, *S. kudriavzevii* strains ZP591 and IFO1802 exhibit the highest *CUP1* expression within non-domesticated *Saccharomyces* species, yet are highly sensitive to copper (Fig. 6A). Conversely, the synthetic *S. cerevisiae* × *S. uvarum* hybrid H14A7 (Lairon-Peris et al. 2020), the most copper-tolerant strain in this group, shows the highest *CRS5* expression (Fig. 6B).

To gain deeper insight into gene expression patterns, we collectively analyzed the relative expression profiles of the four genes. This analysis revealed distinct groups of yeasts with different strategies for copper tolerance (Fig. 6C). Group III, which includes several *S. cerevisiae* strains and the closely related species *S. paradoxus* and *S. kudriavzevii*, primarily relied on *CUP1* expression, with comparatively low *CRS5* expression (Fig. 6C). In contrast, Group Ib, comprising species evolutionarily more distant from *S. cerevisiae*, such as *S. arboricola*, *S. eubayanus*, *S. uvarum*, and *S. chiloensis*, showed a predominant reliance on *CRS5* expression over *CUP1* (Fig. 6C). Other groups (Ia and II) exhibited relatively low dependence on *CUP1*, but increased expression of either *CRS5*, *SOD1*, or *TRX2* (Fig. 6C). These findings suggest that in *Saccharomyces* species carrying a single *CUP1* copy, both metallothioneins contribute to copper tolerance, with *CUP1* playing a more prominent role in species closely related to *S. cerevisiae*, and *CRS5* in more distant species. Additional molecular mechanisms beyond metallothioneins likely influence copper tolerance in wild *Saccharomyces* yeasts.

## Discussion

The molecular response to elevated copper concentrations in *S. cerevisiae* has been extensively characterized in laboratory strains derived from the S288C background, such as BY4741. These strains typically carry multiple tandem repeats of the *CUP1* locus on chromosome VIII. However, a comprehensive analysis of natural trait variation across diverse *S. cerevisiae* isolates revealed that S288C-derived strains are highly atypical and strongly diverging from the species-wide average and thus cannot be considered as representative of the whole species (Warringer et al. 2011). The high number of copies of *CUP1* gene, combined with its superior copper-binding capacity and the more robust copper-induced expression of *CUP1* relative to *CRS5* (Jensen et al. 1996), explains its dominant contribution to copper homeostasis in S288C-derived *S. cerevisiae* isolates (Peter et al. 2018; Strope et al. 2015; Warringer et al. 2011). In our study, we observe a clear positive correlation between *CUP1* CNV and copper tolerance in wine-related *S. cerevisiae* yeasts, whereas no such relationship is detected in wild *Saccharomyces* isolates. By contrast, a recent study reported no correlation between *CUP1* CNV and copper fitness in wine *Saccharomyces* strains due to variation in the expression of the sulfite efflux pump *SSU1*, whose overexpression limits intracellular sulfur availability and consequently increases copper sensitivity (Onetto et al. 2023). It is likely that the trade-off between sulfite and copper tolerance is not sufficiently pronounced in the wine yeast strains analyzed in our work to disrupt the correlation between *CUP1* CNV and copper tolerance. Moreover, the enhanced expression of *CUP1* is not always associated with reduced copper-induced oxidative stress. For example, the tolerant strain K1M exhibits ROS levels higher than expected, and BY4743 displays a significantly elevated proportion of dead cells despite its high copper tolerance. Conversely, the most copper-sensitive wine yeasts analyzed, W27 and W46, are *S. cerevisiae* × *S. kudriavzevii* hybrids characterized by a low *CUP1* copy number and expression, coupled with high *CRS5* mRNA levels. Moreover, W27 strain is able to maintain ROS levels low at both 14 and 18 h, probably due to higher *SOD1* and *TRX2* expression levels. The same can account to explain the low percentage of dead cells in this strain compared to BY4743. These results indicate that several mechanisms coexist with *CUP1* amplification and upregulation, together contributing to copper tolerance in *S. cerevisiae* (Fogel and Welch 1982; Hamer et al. 1985; Strope et al. 2015; Warringer et al. 2011).

Interestingly, increased *CUP1* expression correlates with reduced mRNA levels of the *CRS5* metallothionein. At first glance, this appears counterintuitive, as both genes are activated in response to high copper concentrations through the Ace1 transcription factor (Culotta et al. 1994; Thiele 1988; Welch et al. 1989). Previous studies have shown that *CUP1* induction upon copper exposure is more pronounced than *CRS5*, likely due to the presence of three Ace1-binding sites (known as Copper-Regulated Elements, CuREs) in the *CUP1* promoter compared to a single CuRE in the *CRS5* regulatory region (Jensen et al. 1996). A plausible explanation for the negative correlation between *CUP1* and *CRS5* expression is that the multiple CuREs within amplified *CUP1* promoters may sequester Ace1, thereby reducing its availability for *CRS5* transcriptional activation.

*S. cerevisiae* strains and other *Saccharomyces* species from wild environments, presumably unexposed to high copper concentrations, harbor a single copy of the *CUP1* gene and display greater copper sensitivity, although the degree of sensitivity varies among species. Importantly, *CUP1* expression in these strains did not correlate with copper tolerance, indicating that, in the absence of adaptation to copper-rich environments, additional molecular mechanisms beyond Cup1-mediated detoxification contribute to copper homeostasis when *CUP1* dosage remains low. Our comparative expression analysis of *CUP1* and *CRS5* indicates that the predominant use of each metallothionein is species-dependent: species closely related to *S. cerevisiae* primarily express *CUP1*, whereas more distantly related species preferentially express *CRS5*. Previous studies have demonstrated allelic divergence in the copper-regulated transcription factor *ACE1*/*CUP2* between *S. cerevisiae* and *S. uvarum*, involving both *cis*-regulatory elements and coding sequences (Li and Fay 2019). Similarly, differences in promoter structure and metal-binding preferences help explain the stronger copper detoxification capacity of Cup1 compared with Crs5 in laboratory *S. cerevisiae* (Jensen et al. 1996; Palacios et al. 2011; Pagani et al. 2007). However, the reasons why different *Saccharomyces* species rely on Cup1 or Crs5 for copper detoxification remain unclear.

Recent evidence indicates that *S. cerevisiae*, unlike many other yeast species, has evolved specialized strategies to thrive in copper-rich environments (Fay et al. 2026; Longan and Fay 2024). Nevertheless, the molecular basis of copper detoxification in *Saccharomyces* species remains only partially understood, as Cup1 accounts for just a fraction of the observed phenotype (Strope et al. 2015; Warringer et al. 2011)*.* Notably, despite the downregulation of high-affinity copper transporters at the plasma membrane, *S. cerevisiae* does not fully restrict copper uptake as extracellular concentrations increase (Kim et al. 2023) and, in contrast to many microorganisms, lacks copper-exporting ATPases that remove excess copper from the cell, a trait likely shared across the *Saccharomyces* genus. Instead, copper toxicity is mitigated primarily through the Ace1-mediated induction of metallothioneins and superoxide dismutase, which buffer and detoxify cytosolic copper (Gross et al. 2000), as well as Fet3, which modulates the oxidation state of copper (Shi et al. 2003). As shown in this study, the relative contributions of *CUP1* and *CRS5* metallothioneins appear to vary among species. Although most cytosolic copper associates with Cup1 (Kim et al. 2023) and likely Crs5, additional factors such as amino acid metabolism, particularly histidine and tryptophan (Jo et al. 2008; Pearce and Sherman 1999), and glutathione (Freedman et al. 1989) contribute to copper tolerance. In *S. cerevisiae*, vacuoles serve as major subcellular reservoirs for copper buffering and storage, thus contributing to copper tolerance (Gerstein et al. 2015). Excess copper is transported into the vacuole by an as-yet unidentified mechanism and mobilized when required (Rees et al. 2004). Consistent with this, mutants defective in vacuole biogenesis or acidification exhibit impaired copper detoxification (Eide et al. 1993; Jo et al. 2008; Szczypka et al. 1997). Moreover, *S. cerevisiae* can tolerate high copper levels by depositing copper salts in the cell wall through a process dependent on the RNA-binding protein Slf1, as mutants lacking this protein are copper-sensitive, and its overexpression increases copper tolerance (Ruta and Farcasanu 2021; Schenk et al. 2012; Yu et al. 1996).

Copper also localizes to mitochondria via Cup1 and Sod1 intermembrane space proteins, cytochrome oxidase, and a non-proteinaceous pool within the matrix (Cobine et al. 2004; Kim and Lindahl 2023). Temperature adaptation is widely recognized as a major driver of *Saccharomyces* speciation. Within the genus, several species, such as *S. eubayanus*, *S. uvarum*, and *S. chiloensis* are characterized as cryotolerant, whereas others, including *S. cerevisiae* and *S. paradoxus* are typically considered thermotolerant (Peris et al. 2023). Adaptation to low temperatures has also been linked to mitochondrial genomic variation, particularly to specific haplotypes of the *COX1* gene, which encodes cytochrome c oxidase (Li et al. 2019). As mentioned above, cytochrome c oxidase is a key copper‑dependent enzyme, suggesting that mitochondrial differences may influence species‑specific copper homeostasis pathways.

**Fig. 7:**
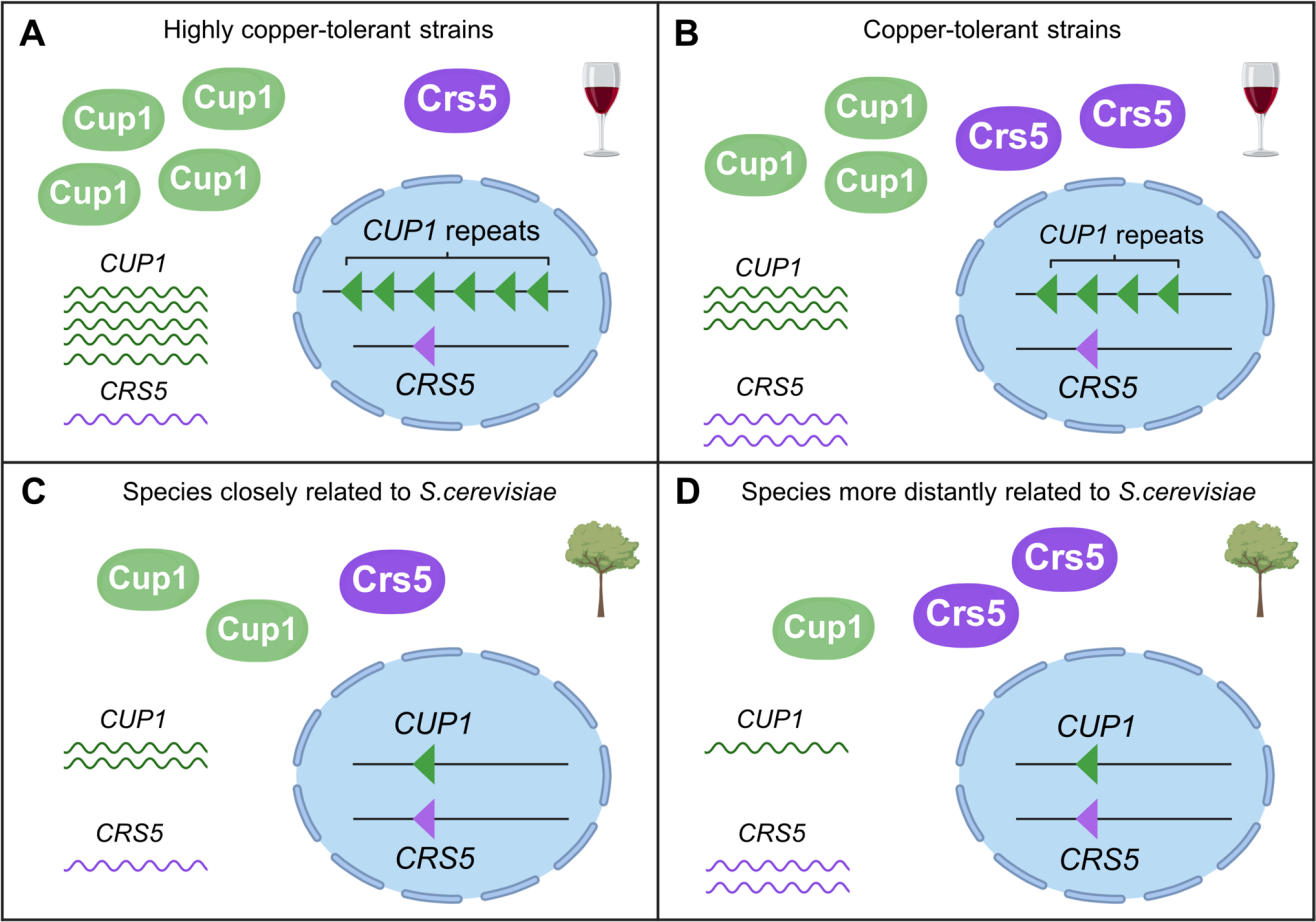
Contribution of the metallothioneins Cup1 and Crs5 to copper tolerance across the *Saccharomyces *genus. **A** Highly copper-tolerant strains harbor an increased number of tandem *CUP1* copies and show strong *CUP1* expression in response to excess copper. **B** Wine *S. cerevisiae* strains and hybrids with lower copper-tolerance carry fewer *CUP1* copies and display increased *CRS5* expression. **C** Non-domesticated *Saccharomyces* strains closely related to *S. cerevisiae*, such as *S. paradoxus* and *S. kudriavzevii*, contain only a single *CUP1* copy but still express *CUP1* at higher levels than *CRS5*. **D** More distantly related *Saccharomyces* species including *S. arboricola*, *S. eubayanus*, *S. uvarum*, and *S. chiloensis*, express *CRS5* more strongly than *CUP1* under high copper conditions. Created in BioRender and licensed under CC BY 4.0. Puig, S. (2026) https://BioRender.com/fri5fqm

In summary, our study demonstrates that alternative copper‑buffering systems, such as the Crs5 metallothionein, may play a proportionally more important role in copper detoxification in wine and wild yeast strains and species carrying only a single *CUP1* copy (see Fig. 7). These findings highlight copper tolerance as a multifactorial trait that remains far from fully understood. Further studies will be required to elucidate the relative contribution and interplay of these mechanisms in shaping copper resistance.

## Supplementary Information

Below is the link to the electronic supplementary material.

ESM 1 (456 KB PDF)

ESM 2 (74.3 KB XLSX)

## Data Availability

The primary datasets used in the preparation of this manuscript, along with their analyses, are openly available in Digital CSIC http://hdl.handle.net/10261/418454.
