# Supplementary material for: Divergent metallothionein strategies underlie copper tolerance in Saccharomyces species relevant to winemaking
Source: Appl Microbiol Biotechnol. 2026 May 21;110:213. doi: 10.1007/s00253-026-13869-z (PMC13369610; doi:10.1007/s00253-026-13869-z)
Supplement: Supplementary file 1 [file 253_2026_13869_MOESM1_ESM.pdf]

Applied Microbiology and Biotechnology

Supplementary material

Divergent metallothionein strategies underlie copper tolerance in *Saccharomyces* species relevant to winemaking

Raquel Sorribes-Dauden[a,*], David Peris[a,b], María Teresa Martínez-Pastor[a,c], Sergi Puig [a,*]

[a] Departamento de Biotecnología de Alimentos, Instituto de Agroquímica y Tecnología de Alimentos (IATA), Consejo Superior de Investigaciones Científicas (CSIC), 46980. Paterna, Valencia, Spain.
[b] FunGIALab, Department of Biosciences, University of Oslo, 0371, Oslo, Norway.
[c] Departamento de Bioquímica y Biología Molecular, Universitat de València, 46100, Burjassot, Valencia, Spain.

* To whom correspondence should be addressed:
Sergi Puig (Email: spuig@iata.csic.es) and Raquel Sorribes-Dauden (Email: raquel.sorribes@iata.csic.es). CuFeLab, Dept. Biotecnología de Alimentos, IATA-CSIC, Catedrático Agustín Escardino 7, 46980, Paterna, Spain. Tel: (+34) 963 900 022.

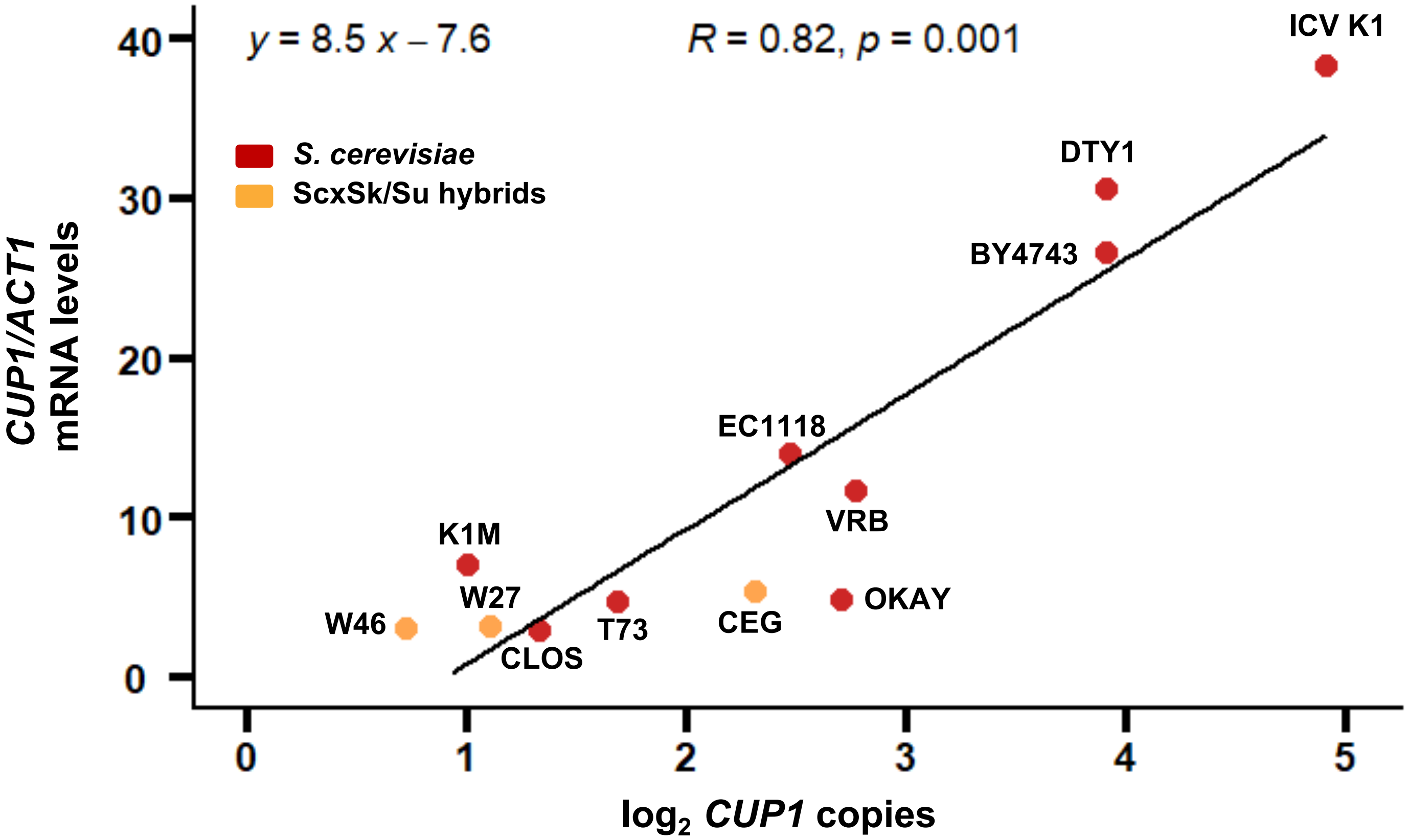

Supplemental Figure S1. Scatterplot analysis of $\log_2$ *CUP1* copies (x axis) and *CUP1* mRNA levels after one hour exposure to 5 mM $CuSO_4$ in yeasts with more than one *CUP1* copy. Data represent the average of three independent biological replicates and is normalized to *ACT1* mRNA levels in the same conditions. Dot colors follow the same code as principal figures. Spearman's correlation equation and coefficient of correlation (*R*) are shown, together with *p*-value (*p*).

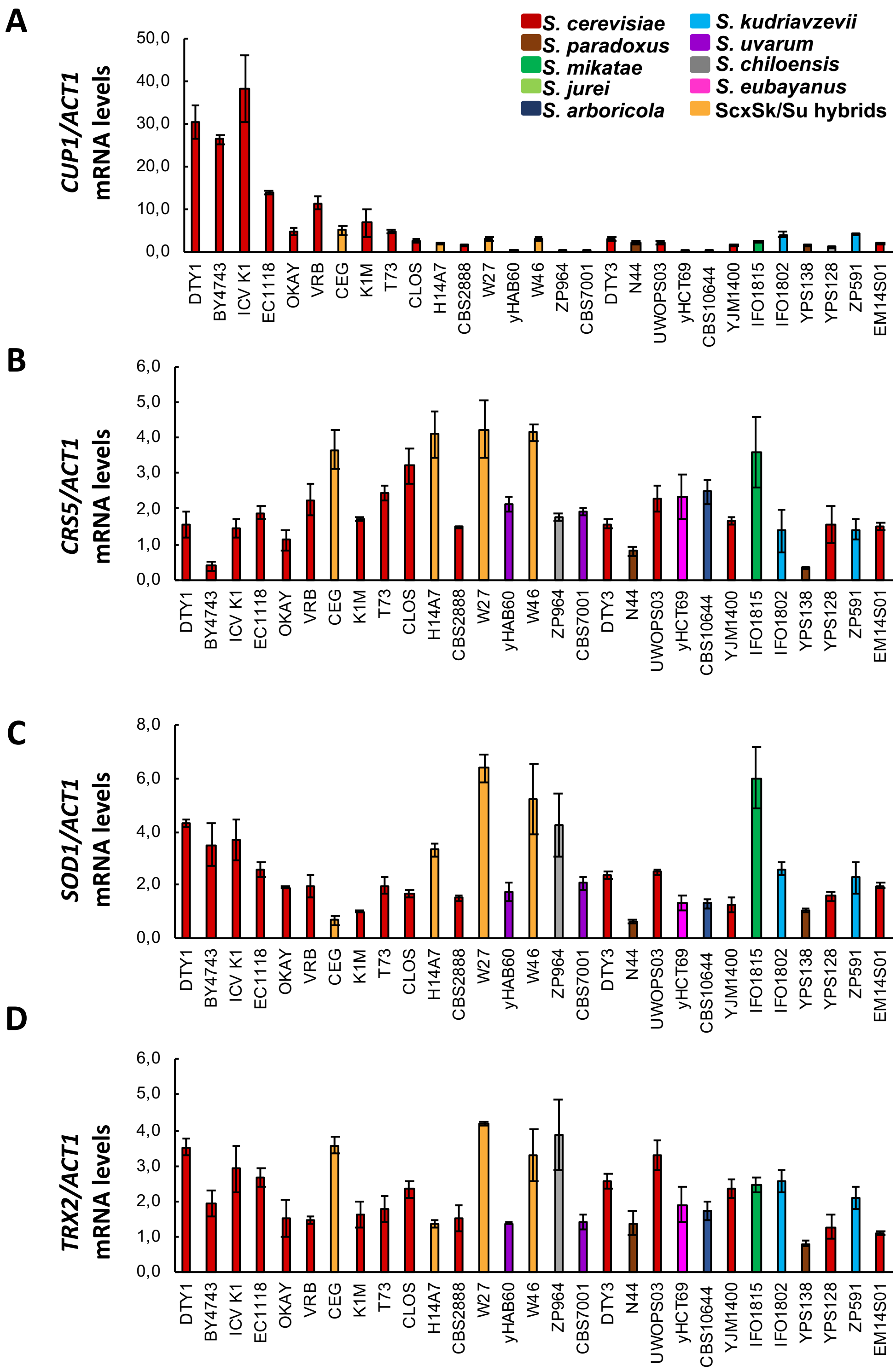

Supplemental Figure S2. Effect of copper exposure on gene expression. *CUP1* (A), *CRS5* (B), *SOD1* (C), and *TRX2* (D) mRNA levels after copper incubation. Bars and errors represent the average and standard deviation of three independent biological replicates, respectively. mRNA levels were normalized to *ACT1* mRNA levels in the same conditions. Strains are ordered according to MIC values and bar colors described species as legend.

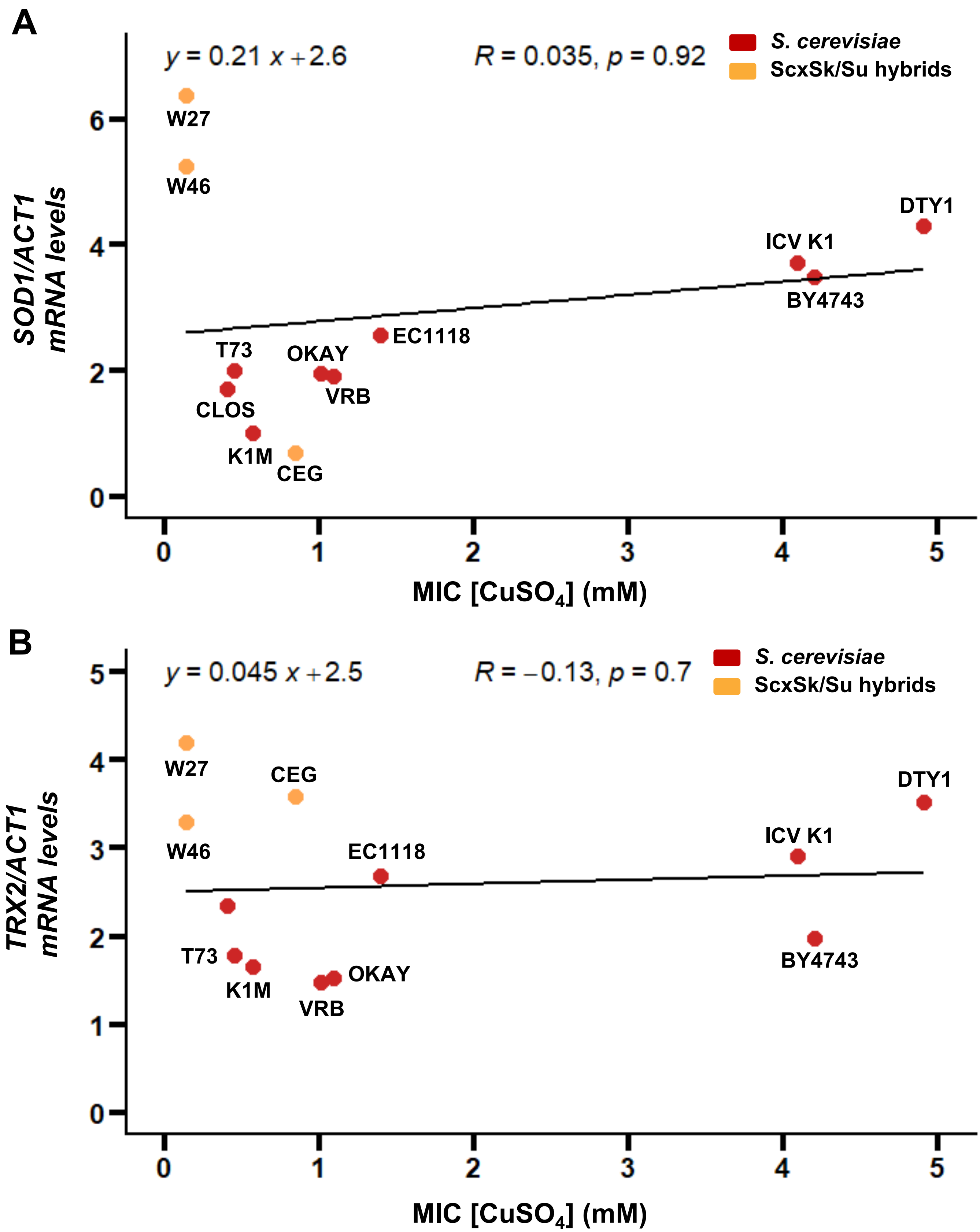

Supplemental Figure S3. *SOD1* and *TRX2* mRNA levels do not correlate to copper tolerance in yeasts with more than one copy. *SOD1* (A) and *TRX2* (B) mRNA levels, normalized to *ACT1*, after copper exposure plotted against MIC (mM). Spearman's correlation equation, correlation coefficient (*R*), and *p*-value *(p)* are shown. Species are indicated by dot colors

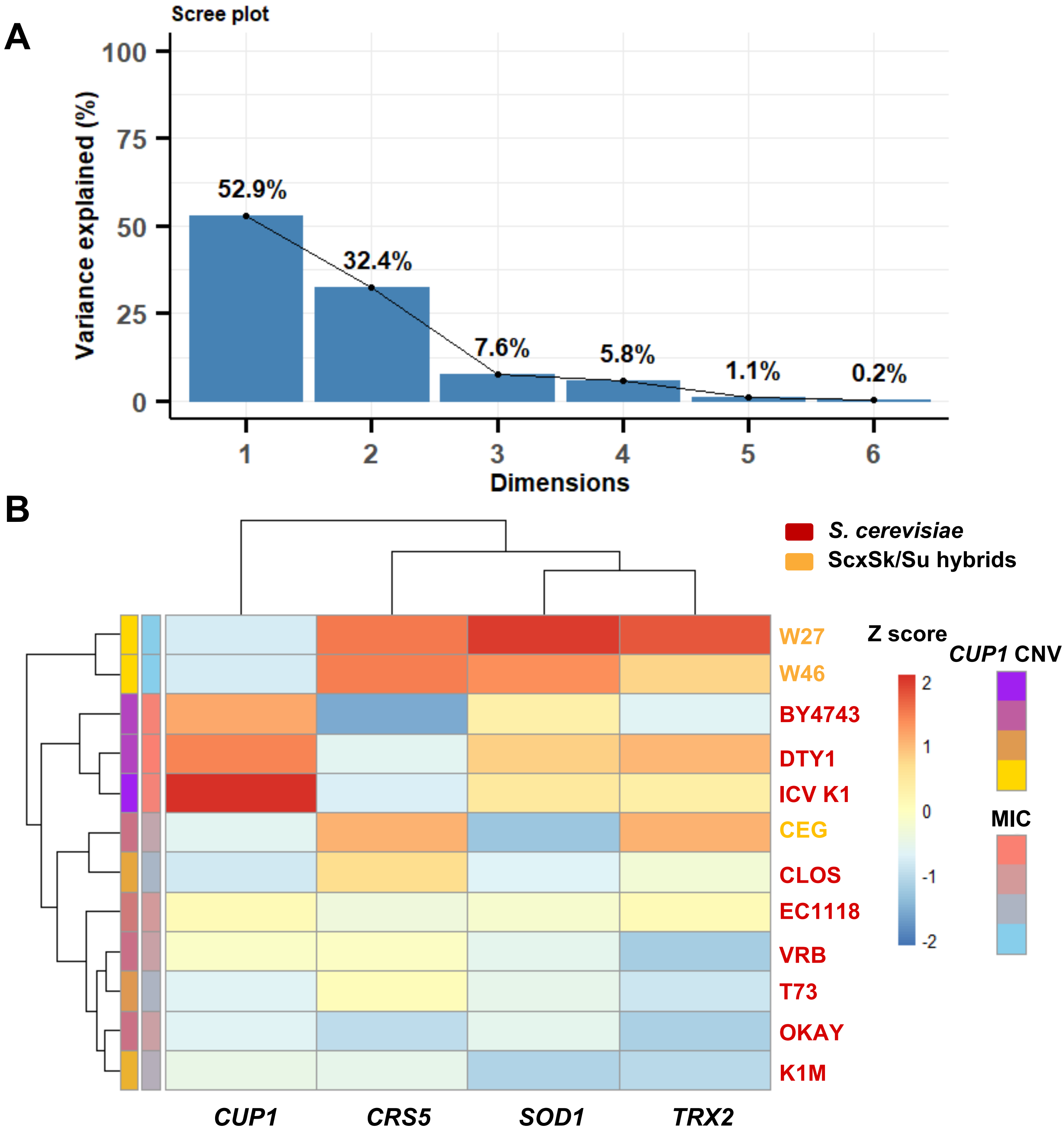

Supplemental Figure S4. In wine strains, copper-tolerance relies on *CUP1* expression whereas copper-sensitive strains depend more on *CRS5* mRNA levels. (A) Scree plot illustrating the proportion of total variance explained by each principal component obtained from the PCA for yeasts with more than one copy of *CUP1*. (B) Heatmap analysis of *CUP1, CRS5, SOD1*, and *TRX2* mRNA levels in strains carrying more than one *CUP1* copy. Hierarchical clustering was performed based on Euclidean distance. Z-score normalization was performed for each gene and high values are shown in red, whereas low values are shown in blue. Tolerance is depicted as $\log_{10}$(MIC), with high values shown in salmon pink and low values in light blue. $\log_2$ (CNV) values are represented in purple (high) and yellow (low).

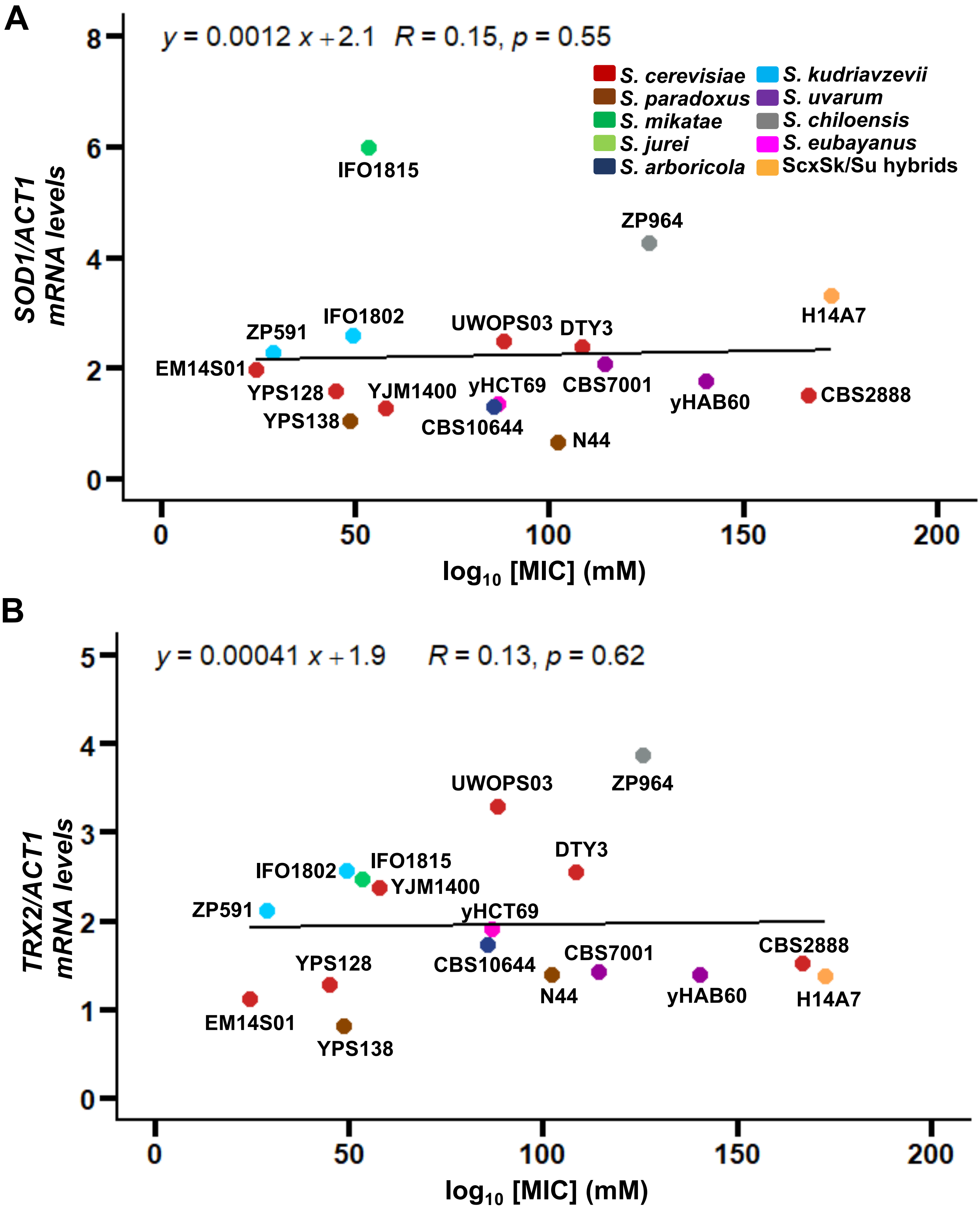

Supplemental Figure S5. *SOD1* and *TRX2* mRNA levels do not correlate to copper tolerance in strains with one *CUP1* copy. *SOD1* (A) and *TRX2* (B) mRNA levels after copper exposure normalized to *ACT1* mRNA in the same conditions, versus MIC in mM. Spearman's correlation equation and correlation coefficient (*R*) are shown, together with *p*-value (*p*). Species are denoted as colored dots according to the legend.
